# Roles of microbial interactions in determining the establishment and function of synthetic consortium inoculants for soil applications

**DOI:** 10.1093/ismejo/wrag050

**Published:** 2026-03-11

**Authors:** Yinuo Xu, Shilva Shrestha, Qing Sun, Ying Wang

**Affiliations:** Department of Soil and Crop Sciences, Texas A&M University, College Station, TX 77843, United States; Department of Environmental Health and Engineering, Johns Hopkins University, Baltimore, MD 21218, United States; Artie McFerrin Department of Chemical Engineering, Texas A&M University, College Station, TX 77843, United States; Interdisciplinary Graduate Program in Genetics and Genomics, Texas A&M University, College Station, TX 77843, United States; Department of Soil and Crop Sciences, Texas A&M University, College Station, TX 77843, United States

**Keywords:** synthetic consortium inoculants, microbial interactions, native soil communities, establishment, function, invasion, resistance and resilience

## Abstract

Synthetic microbial consortium inoculants are emerging nature-based solutions for promoting sustainable agriculture and mitigating environmental challenges. However, despite promising results in simpler lab-scale trials, many inoculants fail to establish or perform satisfactorily in field conditions. One most critical yet least understood factor influencing inoculant effectiveness is the complex microbial interactions, both within consortium inoculants (“within-community” interactions) and between consortium inoculants and native soil communities (“cross-community” interactions). Here, we first discuss major negative and positive “within-community” interactions and highlight the importance to design consortium inoculants with positive interactions for improved stability and functionality. We then examine the bidirectional “cross-community” interactions once introducing consortium inoculants to soils. Soil native communities often create strong resistance to the invasion of inoculants. We discuss major drivers controlling the invasibility of native communities and various strategies increasing the invasiveness of consortium inoculants. We then discuss how consortium inoculants can reshape native communities, with implications for long-term ecosystem resilience and functioning. We propose future research efforts including advancing strategies for harnessing natural species from relatively untapped soil reservoirs and using high-throughput interaction profiling with multi-omics and computational tools to build compatible synthetic consortia with desirable functions; leveraging positive interactions and prebiotics to facilitate inoculant establishment; and assessing fully soil functional resilience over longer terms, including recognizing the importance of rare keystone taxa. By integrating with ecological theory, this review provides a comprehensive insight into microbial interactions to advance the design, application, and monitoring of synthetic consortium inoculants for enhancing soil health and ecosystem sustainability.

## Introduction

As global issues such as climate change, biodiversity loss, and environmental degradation persist, nature-based solutions are increasingly recognized for their potential to restore ecosystem functioning and resilience [1, 2]. Soil ecosystems are at the core intersection of agricultural, socioeconomic, and climate problems and have the potential to mitigate many of these environmental problems [3, 4]. One promising approach involves inoculating soils with synthetic microbial communities to harness the beneficial properties of diverse microorganisms [5].

Synthetic communities are rationally designed microbial consortia that can be inoculated to soils for various purposes, including climate mitigation, carbon sequestration, plant growth promotion, and bioremediation to improve overall ecosystem functioning [6–10]. The design of synthetic consortia generally follows two approaches: the top–down design typically enriches a naturally simplified community through repeated selection under defined conditions for specific functions, whereas the bottom–up design selects individual strains with desired traits and combines them into a defined assembly [11–13]. Compared with single-species inoculants, synthetic microbial consortium inoculants bring diverse species together and can offer potentially higher ecological adaptability and more versatile desired functional outcomes [14, 15]. However, despite promising results in simpler lab-scale (culture tube, pot, or greenhouse) trials, many synthetic consortium inoculants fail to establish or perform satisfactorily in complex field soils [16, 17]. A previous meta-analysis on bacterial consortium inoculants for promoting plant growth has found that more pronounced effects with lower variability were observed in pot/greenhouse experiments than under field conditions [15]. One of the most critical yet least understood factors influencing the effectiveness of consortium inoculants is the complex microbial interactions, both within the inoculant communities (“within-community” interactions) and between the inoculants and native soil communities (“cross-community” interactions).

“Within-community” interactions among members of the synthetic consortia shape the structure, stability, and collective function of the inoculants. The top–down approach often yields synthetic consortia with desired functions but without a mechanistic understanding of the underlying interaction mechanisms, prohibiting further simplification and manipulation for precise applications [11]. By comparison, the bottom–up approach requires identifying candidate strains with targeted functional potentials and then screening for strains that are compatible with each other. Most current traditional methods are far from sufficient, as they primarily focus on the limited numbers of common culturable microbes and often overlook the interaction mechanisms driving community stability and emergent properties. Competitive interactions can significantly decrease microbial growth and disrupt signaling, which can hinder functional performance [18]. In contrast, cooperative interactions, such as nutrient cross-feeding, can enhance the overall metabolic activity of the inoculants [19]. Positive interactions between keystone (species having critical roles in determining community structure and/or performing specific functions) and “helper” species (species promoting others’ growth and/or activities) may particularly promote community stability and functional performance [20, 21]. Metabolic division of labor allows consortium members to each specialize in a specific task within a complex process to reduce individual metabolic burden [12, 22]. Still, the underlying mechanisms driving dynamic interactions within synthetic consortium inoculants remain elusive, posing a challenge to the optimization of assembling stable consortia with specific functions.

Once introduced into the soil environment, synthetic consortium inoculants encounter complex and dynamic native communities, resulting in bidirectional “cross-community” interactions, with implications for inoculant performance and soil resilience. Native communities can resist the establishment of synthetic consortium inoculants through niche preemption, superior resource acquisition, and phylogenetic relatedness [23–25]. This is likely among the main reasons why synthetic consortium inoculants often fail in the field, despite successful performance in much simpler laboratory or greenhouse conditions. Little is known about the long-term fate and activities of inoculants in soil communities. Further, synthetic consortium inoculants can act as microbial invaders, introducing new functional traits and altering the biomass, structure, and diversity of native communities [26, 27]. Resistance and resilience of the native communities play critical roles in determining both the functional outcomes of the synthetic inoculants and their potential long-term impacts on soil ecosystems. Even though technical advances have enabled assessment of inoculant effects on soil microbial community biomass and composition, the underlying mechanisms and the resulting effects on ecosystem functioning remain poorly understood [26–28]. Addressing these knowledge gaps is crucial for understanding how synthetic consortium inoculants can establish in native communities and influence long-term ecosystem performance.

Here, we discuss key “within-community” and “cross-community” interactions that shape synthetic consortium inoculant success. Within the synthetic consortium inoculants, we discuss major interactions driving internal stability and functional compatibility. Beyond the inoculants themselves, we discuss the complex dynamics of how consortium inoculants can invade and interact with soil native communities, particularly by leveraging insights from ecology theories on invasion and resilience. The “cross-community” interaction outcomes are largely governed by the invasiveness of the inoculants and the resistance and resilience of native communities, ultimately influencing long-term establishment and ecosystem effects. We summarize a list of >100 studies on synthetic consortium inoculants in soil applications, to highlight major gaps in our understanding of microbial interactions in determining inoculant success (Supplementary Table S1). We propose further research to advance the mechanistic understanding of these complex interactions to harness the full potential of synthetic consortium inoculants for achieving climate mitigation, sustainable agriculture, and resilient ecosystems.

## “Within-community” interactions of synthetic consortium inoculants

**Figure 1:**
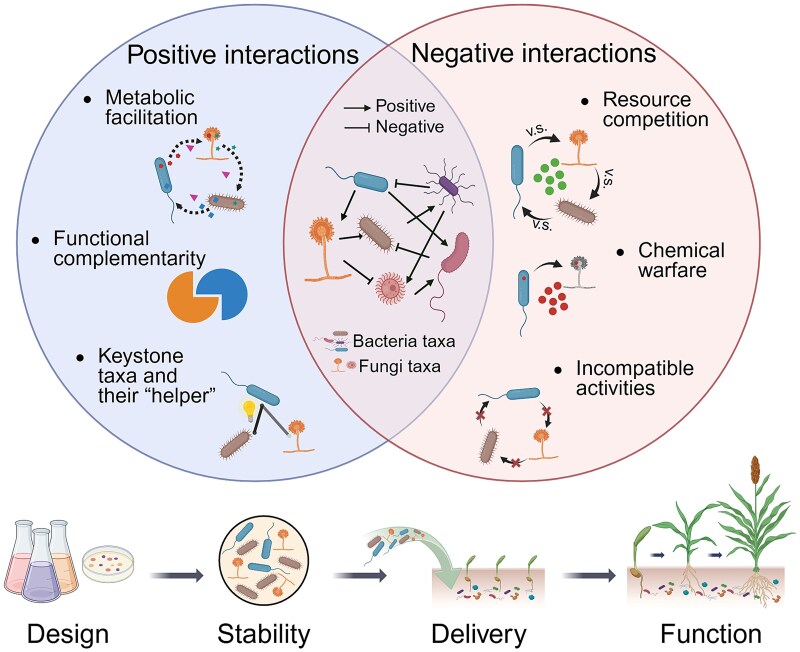
Effects of “within-community” interactions on structural stability and functional effectiveness of synthetic consortium inoculants for soil applications. Potential consortium members (middle panel) including bacterial and fungal taxa may engage in both positive and negative interactions. Positive interactions (left panel), such as metabolic facilitation, functional complementarity, and keystone with helper species, can enhance community stability and maintain functional performance. In contrast, negative interactions (right panel), including resource competition, chemical warfare, and incompatible activities, may destabilize community structure and reduce functional effectiveness. The design of consortium inoculants should prioritize positive “within-community” interactions to enhance inoculant stability, functional performance, and persistence. Created in BioRender. https://BioRender.com/tbw5lqa.

Central to the success of microbial consortium inoculants are the “within-community” interactions that regulate metabolic exchange, assembly, and compatibility, ultimately determining community stability and functioning. A recent meta-analysis showed that compared with single-species inoculants, consortium inoculants have better inoculation effects for biofertilization and bioremediation in living soils [15]. This highlights the importance of understanding the mechanisms, in particular, how the complex microbe–microbe interactions within synthetic consortium inoculants contribute to their effectiveness (Fig. 1).

Both positive and negative interactions are naturally widespread within microbiomes [29, 30], balancing the complex dynamics that shape the stability and functionality of consortium inoculants. Negative interactions among members can take place when one species inhibits another species’ growth through nutrient competition and chemical warfare, decreasing the productivity of the entire community [18, 30]. Additionally, incompatible microbial biochemical activities within inoculant communities can reduce functional effectiveness. For example, co-inoculation of *Bacillus subtilis* 101 and *Azospirillum brasilense* Sp245 did not improve plant biomass, although each microbe individually enhanced plant growth [31]. This negative outcome of the dual inoculation was attributed to potential interference between independent signaling pathways, resulting in incompatibility between the microbes in the mixture. To avoid undesirable negative interactions, it is crucial to carefully select compatible community members when formulating consortium inoculants, enabling the development of stable communities that thrive through positive relationships.

Positive interactions, such as metabolic cross-feeding and exchange of public goods, can enhance community assembly and productivity [19, 32]. Previously, it was thought that competitive interactions are more dominant in microbial communities [33], but recently, it has been shown that positive interactions can occur commonly [32]. This prevalence of positive interactions, particularly the growth–promoting effects on nongrowing strains, was likely due to the secretion of carbon source–degrading enzymes or incompletely oxidized metabolites by the facilitators. Other studies have shown that plant growth–promoting bacteria and arbuscular mycorrhizal fungi (AMF) can work synergistically in the rhizosphere, where carbon–rich exudates from AMF can reshape bacterial communities and bacteria can mineralize organic phosphorus for AMF [34, 35]. These results highlight that positive interactions within the community should be treated as criteria for assembly during predictive consortium design, rather than being inferred retrospectively from functional outcomes. By screening for species with cooperative metabolic interactions and enhancing their compatibility through supportive metabolites, it is possible to strengthen internal community cohesion.

Positive interactions among microbial species in consortium inoculants can also enhance functional performance by enabling members to compensate for traits lacking in others and support key ecological roles. For example, promoting metabolite cross-feeding for the division of labor, where different species perform specialized tasks and become interdependent, can enable simultaneous lignin depolymerization and upgrading [36]. Similarly, in another study, five native bacteria isolated from *Nicotiana attenuata* root functionally complement each other *in vitro* [37]. As a consortium, they engaged in synergistic interactions and protected host plants by their ability to combine different modes of biocontrol action *in planta*.

Positive interactions between keystone taxa and their “helper” benefit the structural and functional performance of synthetic consortium inoculants. Keystone taxa are the main drivers for community composition and function, irrespective of their abundance [20], whereas “helper” species have been reported to promote the growth and/or functional gene expression of keystone members [38, 39]. For instance, the removal of keystone species *Enterobacter cloacae* led to the complete breakdown of an entire simplified synthetic bacterial community [40], highlighting the crucial role of keystone species in sustaining community stability. Other studies further illustrate how “helper” species support beneficial functions in the community. For example, it was found that “mycorrhiza helper bacteria” stimulated AMF hyphae growth by releasing gaseous volatiles, leading to improved root colonization [41]. Another example showed that two “helper” non-nitrogen-fixer species exhibited strong respiratory metabolism, thereby creating a micro-oxic environment that benefited the nitrogen-fixation capabilities of two diazotrophs [42].

Overall, an increasing number of studies have focused on applying synthetic consortium inoculants for specific beneficial functions, yet fewer have focused on examining the underlying “within-community” interactions that govern consortium stability, persistence, and functional performance under realistic conditions. Although general ecological principles, such as competition, facilitation, division of labor, and keystone effects, are increasingly understood in microbial communities, applying these mechanistic understandings to predictive engineering for synthetic consortium inoculants remains limited [11]. Among the 104 studies of consortium inoculants in soil we collected, only 22 studies explicitly examined “within-community” interactions, primarily through coculture assays to confirm nonantagonistic or synergistic interactions among isolates before consortium assembly; two additional studies inferred interactions indirectly based on inoculation outcomes (Supplementary Table S1). The collective activity of a consortium may not simply be the sum of activities of its individual members [12, 30]. Empirical evidence demonstrates that positive “within-community” interactions emphasize the importance of community cooperation in enhancing the stability and functionality of synthetic consortium inoculants [43, 44], whereas negative interactions such as resource competition can undermine them [45]. As a result, exploring these interactions is essential for understanding the behavior of individual microorganisms within the consortium inoculants and for unraveling the emergent performance of the entire community. Integrated high-throughput experimental and computational frameworks for effectively quantifying and linking interactions to stability and function under variable environmental conditions are urgently needed [46, 47]. A thorough investigation of “within-community” interactions will benefit not only in designing microbial consortia with targeted functions [11, 12] but also in developing compatible formulations to ensure the viability and stability of inoculants during stages including production and storage prior to their soil applications [48, 49].

## “Cross-community” interactions between synthetic consortium inoculants and native microbial communities

**Figure 2:**
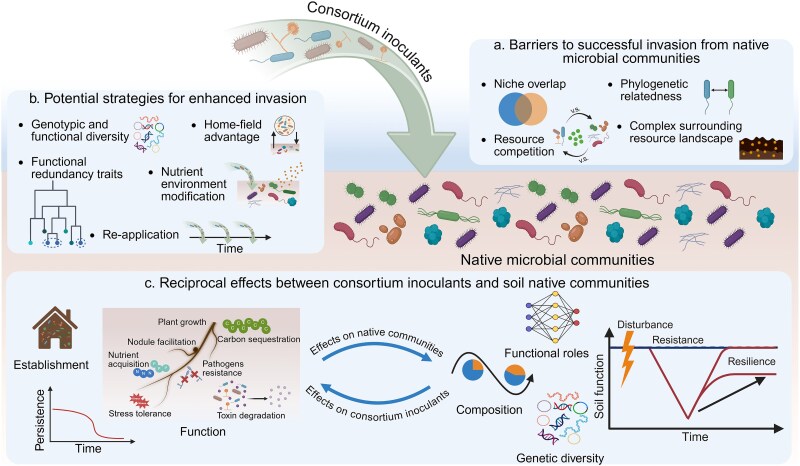
Effects of “cross-community” interactions on both consortium inoculants and soil native communities. (a) Native communities act as barriers to the invasion of consortium inoculants through mechanisms such as niche overlap, resource competition, phylogenetic relatedness, and complex interactions with surrounding resource landscape. (b) Consortium inoculants can potentially increase their invasiveness by strategies including increasing genotypic and functional diversity, functional redundancy, home-field advantage, modifying nutrient environment, and re-application. (c) Following soil applications, “cross-community” interactions have reciprocal effects on consortium inoculants and soil native communities. Strong native resistance may suppress the establishment and persistence of consortium inoculants, affecting their functional performance (such as plant growth promotion, nodulation, nutrient acquisition, stress tolerance, soil carbon sequestration, pathogen suppression, and toxin degradation). Reciprocally, consortium inoculants may influence native communities by shifting taxonomic composition, altering rare and dominant taxa with different functional roles, and modifying genetic diversity. However, the impacts on soil ecosystem functional resilience over long term remain poorly understood. Created in BioRender. https://biorender.com/xvsmy6a.

When synthetic consortium inoculants are applied to soil, they must survive the transition from the often simpler and controlled conditions to the more complex and competitive soil environments, where they interact with native microbial communities. The interactions across the inoculants and native communities (“cross-community” interactions) are inherently dynamic and reciprocal (Fig. 2). Native microbial communities affect the survival, establishment, and functional performance of the consortium inoculants. Reciprocally, the introduced consortium inoculants can act as invaders for native residents, introducing new functional traits and potentially shifting the structure and functioning of native communities.

### Effects of native microbial communities on synthetic consortium inoculants

Native microbial communities often create strong resistance to the invasion of inoculants by occupying ecological niches and outcompeting them for resources. Native communities with higher diversity, especially those containing related species, can increase niche overlap with the invading synthetic consortium inoculants, thereby hindering the establishment of inoculants. The biodiversity–invasibility relationship has been witnessed in many microbial invasion studies where more diverse native communities better resisted the invasion of single-species invaders (e.g. *Escherichia coli* and *Pseudomonas putida*) [24, 50–52]. In one study involving multi-species invaders, litter-born microbial consortia were only able to successfully invade soil when the diversity of the soil microbial community was low [53]. Besides diversity, another compelling example comes from a study on *P. putida* KT2440 as an invader [25]. Its invasion success was strongly suppressed by the presence of a closely related *Pseudomonas* species in the resident community, suggesting that phylogenetic relatedness can intensify competition for similar resources and enhance community resistance to inoculant invaders.

The invasibility of native soil communities by synthetic consortium inoculants is determined not only by the composition of native communities but also by the interactions within the native communities and the surrounding resource landscape. One study found that the biodiversity–invasibility relationship is particularly pronounced in complex resource environments, where niche partitioning enables functionally dissimilar residents to fully occupy ecological space, thus limiting opportunities for single-species invaders [51]. Similarly, a modeling study of microbial interactions showed that invasions caused by single-species invaders were less successful when interactions among resident members were mainly facilitation rather than inhibition, likely due to fewer unoccupied niches available for the invaders [54]. In another work using standardized soil resident communities, the establishment of three *Pseudomonas* single-strain inoculants was found to be limited not only by competition for available nutrient niches but also by inadvertent facilitation of resident growth through metabolite sharing [55]. Together, these findings underscore that native microbial communities can resist invasions through multiple mechanisms. Such ecological barriers are inevitable and highlight the inherent difficulty of establishing synthetic inoculants in complex natural soils [56]. However, many current mechanistic insights into microbial invasion resistance are derived from single-invader experiments, where competitive interactions and niche-based exploration can be more readily isolated [24, 50–52, 55, 57]. More research is needed to examine how these resistance mechanisms may apply to consortium invaders.

Synthetic consortium inoculants can potentially increase their invasiveness through various strategies. For example, even though the biodiversity–invasibility relationship suggests that diverse native communities can resist invasion, higher diversity of the consortium inoculants may also help them overcome native resistance [44, 58]. When consortium inoculants are composed of multiple species, they could enhance community persistence and performance, with diversity promoting establishment and redundancy providing resilience under variable conditions. Specifically, higher genotypic and functional diversity would allow inoculants to occupy wider niches more effectively and increase the probability that at least one member could have higher fitness and escape competitive exclusion [57–59]. Additionally, functional redundancy can enhance robustness by buffering against environmental fluctuations or the loss of functional taxa [60, 61].

It has been shown that inoculants containing native species or applied in their “home” sites are more effective in delivering their functions [27, 62]. This home-field advantage contrasts with the findings that inoculants containing similar species to those in native communities have less invasion success, as discussed above [25]. Adding to the complexity is another study showing that inoculants with higher similarity to resident soil communities caused a greater shift in resident community structure but led to lower biofertilizer effectiveness [63]. This study employed top–down enriched microbial communities rather than bottom–up assemblies, making it difficult to characterize whether the conflicts were driven by competition or other uncharacterized interactions. As a result, the mechanistic basis underlying the relationship between similarity, community restructuring, and functional performance remains unresolved. These mixed results highlight the importance of unraveling the complex “cross-community” interactions to mechanistically understand and predict inoculant establishment and functioning.

Because microbial interactions heavily depend on the nutrient resources, modifying the nutrient environment offers a promising strategy to facilitate the establishment of inoculants. For instance, adding promoting components (e.g. prebiotics) to selectively feed the inoculants can enhance cooperative interactions or increase competitiveness of inoculants over native communities. Previously, prebiotics containing key rhizosphere metabolites were shown to enhance carbon metabolism-associated pathways and favor the growth of synthetic consortium inoculants, reducing the ecological niche for pathogenic bacteria to survive and thereby indirectly lowering the disease incidence in tomato plants [64]. Additionally, selective nutrient niches could be designed to promote inoculant proliferation and functional performance [9]. For example, the use of phosphite as a selective nutrient source supported the growth of a toluene-degrading bacterium inoculant *P. veronii* in a model soil system [65]. Resource dynamics can also influence inoculant success. Studies with single-species inoculants demonstrated that shifts from low to high resource availability, such as during resource pulses, can enable inoculants to take advantage of it and thrive. For instance, single-species *E. coli* O157:H7 inoculant was found to utilize resource pulse and outcompete the resident community, allowing the population to regrow up to four log units [50]. However, whether synthetic consortium inoculants can use similar strategies remains unclear.

Re-application strategies may enable inoculants to compete successfully with the native residents, maintaining inoculant persistence and long-term functional efficiency. Higher propagule pressures (i.e. the frequency and quantity of invaders) can enhance the probability of invasion, suggesting repeated inoculations can improve establishment success [66]. Previously, four-time inoculation of single-strain *P. fluorescens* B177 led to modest improvement in its survival and strongly increased soil nitrate content in soil microcosms [23]. By comparison, in another work involving consortia of mixed strains, the positive effect of repeated inoculation was only observed in one out of six consortium inoculants tested [67]. However, in two studies involving the same inoculants containing phosphate-solubilizing and nitrogen-fixing bacteria, the benefits of repeated inoculation were only observed in one study [68] but not in the other [69]. These mixed findings may be explained by previous model predictions suggesting that the outcome of repeated invasions depends on factors including relative competitive abilities of the invaders vs. residents, as well as diversity of the invaders [58]. This highlights that repeated inoculations alone are insufficient to ensure establishment or functional reliability of synthetic consortia without a clear understanding of the underlying “cross-community” interactions.

Overall, mechanistic research on how synthetic consortium inoculants successfully establish in soil systems remains limited, partly due to the difficulties in tracing the fate and behavior of individual consortium members once introduced into complex native communities. Among the 104 consortium inoculant studies we compiled, only 21 studies have attempted to track inoculant survival in soil over time (Supplementary Table S1). Existing sequencing technologies often lack the resolution and specificity needed to distinguish inoculated strains from closely related native taxa at species and strain levels [70, 71]. Additionally, most existing studies have focused on simplified experimental systems that may not fully capture the complexity of real-world soil interactions; consequently, when consortium inoculants fail to deliver the expected functions in the field, the underlying reasons often remain elusive. This highlights the importance of a deeper understanding of how native soil microbial communities inhibit or facilitate consortium inoculant establishment and functional performance. Current research has primarily focused on negative interactions such as competition, while the potential positive effects of native communities remain poorly understood. For the successful inoculation into native communities, designing inoculants that can engage in positive interactions with the residents would be advantageous. *In silico* modeling has predicted that when the native communities facilitate the invader, the resistance is weakened and the chance of invasion is increased [54]. Elucidating the mechanisms by which inoculants escape from suppression or even benefit from native communities will allow for a more successful application of synthetic consortium inoculants in real soil systems.

### Effects of synthetic consortium inoculants on native microbial communities

When synthetic consortia are inoculated into the natural environments, they can reshape native microbial communities not only by shifting taxonomic composition and functional roles but also by altering the genetic diversity of microbial communities. A previous review found that 86% of 108 studies published up to 2019 reported some effects of microbial inoculants on soil microbial communities [28]. A more recent meta-analysis showed that microbial inoculants did not alter soil microbial diversity but induced major changes in microbial biomass and community structure [27]. However, although both review papers noted that there are different inoculation types—such as bacterial vs. fungal vs. a mixture of both—neither paper separately evaluated the effects of single-strain vs. consortium inoculants. In our compiled 104 studies of consortium inoculants in soil, 67 studies have examined the impacts on native microbial communities but primarily focused on changes in community composition, highlighting a major gap in evaluating the changes in community activities (Supplementary Table S1).

Inoculation can differently alter abundant and rare taxa in native communities. Microbial communities are typically structured such that a small fraction of taxa are abundant, while a majority persist at low abundances as rare taxa [72]. Moreover, 1.5%–28% of microbial taxa are estimated as “conditionally rare,” which are typically present at low abundance but can occasionally become dominant under certain conditions [73, 74]. Although often overlooked, these rare taxa may play “keystone” roles in shaping native microbial community stability and driving multiple ecosystem functions [75, 76]. For instance, following the inoculation of *E. coli* O157:H7 to sediment soils, the rare bacterial taxa showed enhanced stability against invasion compared to the abundant taxa [77]. When examining responses of native soil communities to recurring inoculations of three different consortia, the relatively rare taxa were found to have higher diversity than the abundant taxa and to have increased relative abundances after four times of inoculation at an interval of 45 days [68]. In another study testing consortium inoculants containing one to eight *Pseudomonas* strains, inoculant richness correlated with inoculant establishment and effects on soil resident bacterial communities, resulting in more even communities and increased overall diversity and rare taxa relative abundance [44]. This increase in rare taxa abundance could be because inoculants directly inhibited (e.g. via chemical warfare) dominant taxa or had stronger competitive effects on the more abundant taxa to reduce competitive exclusion of rare taxa. Regardless of the exact mechanism, these shifts in the resident communities, rather than the inoculants themselves, were attributed to contributing to the positive plant growth promotion. These findings suggest that rare taxa play an important role in mediating the outcomes of microbial inoculations, even though they have often been overlooked [23]. Their responses may influence whether introduced consortia successfully establish, alter competitive dynamics with abundant native taxa, or trigger shifts in functional processes. Thus, considering the role of rare taxa is critical for understanding and predicting the long-term ecological impacts of synthetic consortia in soil systems.

Native bacterial and fungal communities can vary their responses to microbial inoculations. For example, the effects of four times of inoculation at an interval of 45 days on rare and abundant taxa differed between bacterial and fungal communities in soil [68]. Specifically, the rarer bacteria and more abundant fungi in the native community were most affected by inoculation. More broadly, a recent meta-analysis of 335 studies revealed that microbial inoculants can significantly alter the structure, but not the diversity, of soil bacterial (based on results of 402 observations) and fungal (based on 119 observations) communities [27]. However, this meta-analysis found that the inoculation only increased the biomass of bacteria (based on 740 observations) but not fungi (based on 433 observations). In contrast, another literature review of 143 cases found that native bacterial communities were more likely to show increased diversity than fungal communities following inoculation [78]. These contrasting findings may reflect variations in study design, environmental factors, or inoculant types, but they also point to fundamental differences between bacterial and fungal communities, such as differences in growth rates, dispersal, and resource acquisition strategies. Studying microbial interkingdom effects after inoculation will be essential for developing a more comprehensive understanding of inoculant impacts on soil microbial communities and their functional outcomes.

Microbial inoculation can influence the genetic diversity of native communities by horizontal gene transfer, which drives genetic adaptations. For instance, high rates of horizontal transfer of the symbiotic island from a single-strain *Bradyrhizobium* inoculant to isolated indigenous rhizobia were observed for up to 7 years after the introduction of soybean and inoculated strains under field conditions [79]. Another field study has demonstrated that 7 years after inoculating the single strain *Rhizobium loti* ICMP3153, 19% of soil isolates shared the same genomic fingerprint, indicating that chromosomal symbiotic genes were transferred from the inoculated strains to nonsymbiotic soil bacteria [80]. These examples with single-strain inoculants illustrate that the influence of a newly introduced microbe on native communities through horizontal gene transfer can last several years. Therefore, it can be assumed that for synthetic consortium inoculants containing multiple engineered or selected strains, genetic exchange driven by horizontal transfer is likely to be more complex and dynamic due to the increased donor diversity and gene transfer pathways. Their ecological consequences on native soil microbial communities require long-term studies for proper assessment.

## Resistance and resilience of soil ecosystems

In the face of environmental changes, soil ecosystems exhibit two key properties: resistance, the ability to withstand disturbance without significant change, and resilience, the capacity to recover structure and function after disturbance [81, 82]. Both properties influence the “cross-community” interactions between synthetic consortium inoculants and native microbial communities, which, in turn, also shape soil resistance and resilience. Resistance can act as a first barrier to the synthetic consortium inoculants' invasion, whereas resilience determines whether introduced consortia can persist or eventually be outcompeted and replaced. As previously discussed, the resistance of native communities is driven by high diversity, strong niche occupation, and competitive interactions, which often limit the initial establishment of inoculants. When synthetic consortium inoculants manage to overcome resistance through various adaptive methods, the native community may transition to resilience, where soil ecosystems begin to recover [83]. Whether the native communities would return to their original state (i.e. the initial composition or function before a disturbance) or transition to a new equilibrium (i.e. an alternative stable state with different but stable composition or function after a disturbance) would be shaped by interactions between the residents and introduced species [84].

Resilience plays a crucial role in determining the long-term fate of synthetic consortium inoculants. Long-term established, diverse native microbial communities generally occupy a wider range of ecological niches, leaving fewer niche spaces available for inoculants, thus making their successful invasion difficult [52, 85]. However, even if consortium inoculants do not persist through coalescence, the transient invasion could still trigger a subsequent transition to an alternative stable state [86], leaving disturbance legacies in the compositional and functional resilience of native microbial communities [83]. For example, a prior inoculation of a single *E. coli* strain failed to establish within 28 days; however, this transient and unsuccessful invasion steered the resident community away from the resources usable by the invader, thus opening opportunities for possible successful future invasions [87]. In contrast, another study comparing single vs. mixed inoculants of plant beneficial bacteria over 180 days with four inoculation events found that the inoculants only transiently altered native community composition in the first 90 days, but finally, the native community showed resilience to the subsequent inoculations in the last 90 days [69].

Although communities can be resilient in terms of their composition, function, or both [83], considerable research has mainly focused on the resilience of community structure to various disturbances, and thus, the corresponding shifts in ecosystem functions remain largely unknown [26]. Additionally, the role of microbial interactions in driving community resilience has not been well understood [83]. Understanding and predicting inoculant outcomes require considering both resistance and resilience in microbial inoculation strategies.

## Opportunities and needs for future research

**Figure 3:**
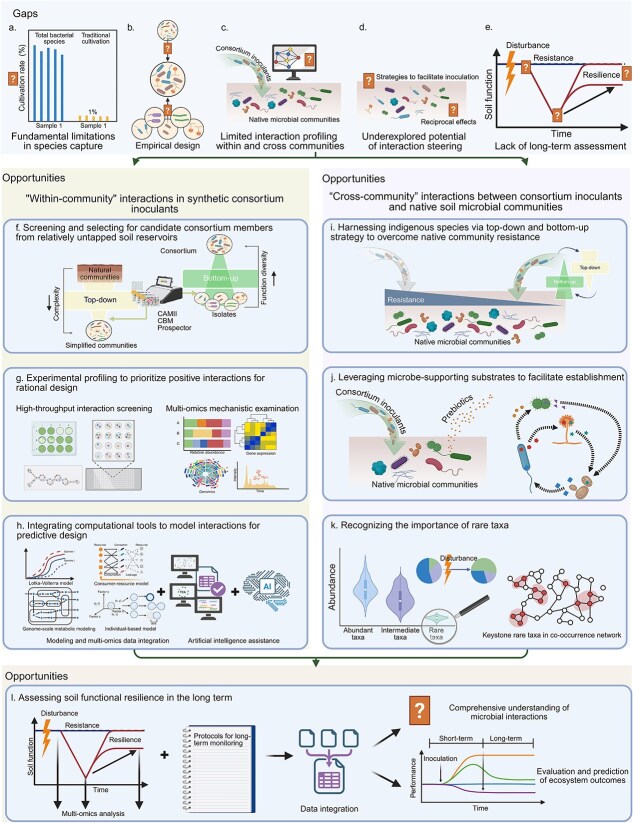
Major gaps and recommended directions for future research on synthetic consortium inoculants for soil applications. Current success of synthetic consortium inoculants is restricted by several gaps. (a) Traditional cultivation methods limit species capture from diverse natural communities for building inoculants. (b) Consortium design remains largely empirical, often without a detailed assessment of “within-community” interactions experimentally or computationally for predictive design. (c) Interaction profiling of both within-consortium inoculants and cross-inoculants and native communities remains insufficient, limiting accurate prediction for inoculant establishment and function, as well as impacts on native communities. (d) The potential of steering interactions to help consortium inoculants overcome native resistance is not well explored, including how consortium design strategies or environmental modification can be beneficial. (e) Long-term ecosystem consequences following inoculation driven by “cross-community” interactions are rarely assessed over time, and functional resilience is poorly understood. To address these, (f) future research should expand species capture using emerging high-throughput culturomics and isolation technologies to harness diverse soil indigenous microbes through integrated top–down and bottom–up strategies for building consortium inoculants. (g) “Within-community” interactions should be experimentally tested using various approaches including high-throughput screening and multi-omics mechanistic examination to prioritize positive interactions for rational design. (h) Modeling and computational tools (including artificial intelligence) should be leveraged to rapidly explore and narrow the large interaction spaces for prioritized experimental validation to guide scalable predictive consortium design. (i) Apply integrated top–down and bottom–up strategies to harness indigenous microbes to design consortium inoculants to facilitate their adaptation and establishment into resident communities. (j) Use microbe-supporting substrates (“prebiotics”) to promote “cross-community” interactions toward facilitation rather than resistance by creating niches and improving competitiveness. (k) Following inoculation, responses of native communities, especially rare taxa, should be considered when evaluating outcomes in community structure and functioning. (l) Together, “within-community” and “cross-community” interactions govern soil ecosystem resistance and resilience. Long-term monitoring with standardized protocols is required to distinguish transient responses from persistent outcomes after soil inoculation. More future work is needed to integrate multi-omics with functional activity analyses to improve understanding of microbial interactions across genetic to community levels for evaluating and predicting long-term ecosystem functioning. Created in BioRender. https://BioRender.com/3ckzpvg.

Unraveling the “within-community” interactions within synthetic consortium inoculants and “cross-community” interactions between inoculants and native communities is crucial for improving inoculant effectiveness in soil applications. To advance synthetic consortia from empirical assembly toward predictive design for improved establishment and function, we propose the following recommendations for future research emphasizing perspectives on microbial interactions (Fig. 3).

### “Within-community” interactions in synthetic consortium inoculants

#### Screening and selecting candidate consortium members from relatively untapped soil reservoirs

One promising direction for designing effective synthetic consortia for soil applications is to harness soil indigenous microbes [62, 88] through integrated top–down and bottom–up strategies. Yet, traditional microbiological cultivation techniques capture only 1% of the total bacterial species [89], and the species that can be cultured do not accurately reflect the true diversity of microbial communities [90]. Consequently, we neglect potentially huge treasure troves of beneficial microbes, particularly from soil environments, limiting our investigation of diverse microbial interactions that drive consortia stability and functionality. Emerging technologies such as reverse-genomics-enabled cultivation of microorganisms can be applied to unlock the isolation, cultivation, and characterization of previously uncultured species [91]. Culturomics by automated microbiome imaging and isolation (CAMII) maximizes morphological diversity with high-throughput acquisition of single-colony genomic data [92]. Similarly, culturomics-based metagenomics (CBM) allows the recovery of novel or specific microbial taxa of interest [93]. Automated systems, such as the Prospector, are enabling high-throughput cultivation, isolation, and screening from complex microbiomes, even those with extremely low abundance [94]. Advancing these technologies will allow us to screen and isolate diverse microbes with synergistic traits in a high-throughput manner, enriching strain resources for future design of consortia with compatible members of desirable functionality.

#### Experimental profiling to prioritize positive interactions for rational inoculant design

Currently, in many studies, consortium members were selected only based on individual functional traits; relatively few studies validated whether candidate members were compatible prior to assembly, with even fewer studies examining interactions in detail under relevant environmental conditions (Supplementary Table S1). This represents a critical limitation because functional complementarity may not necessarily contribute to stable coexistence and collective function at the community level, which requires prioritizing experimental validation of positive “within-community” interactions for consortium design. Interaction screening can be efficiently performed via multiple established methods such as coculture vs. monoculture growth comparison [33], spent media growth experiments [95], and microplate-based coculture devices separating interacting cultures with porous membranes [96, 97]. Additionally, high-throughput microfluidics- and microdroplet-based platforms are greatly improving our capability to systematically screen interactions among large amounts of community compositions across various conditions [98, 99]. Integrating with multi-omics and isotope probing approaches can link specific genes, proteins, and metabolites with functional traits, unraveling complex interaction networks at the molecular level, offering mechanistic insights essential for designing compatible consortia. Future emphasis should be placed on profiling the dynamics of higher-order interkingdom interactions beyond pairwise combinations under soil-relevant conditions. Validating positive “within-community” interactions prior to consortia assembly will help increase internal stability to avoid community collapse after formulation and reduce the risk of reduced functional outcomes due to negative interactions within the consortia during soil applications.

#### Integrating computational tools to model interactions for predictive inoculant design

With the recent advances in high-resolution multi-omics, computational power, and artificial intelligence (AI), modeling should be leveraged to guide and complement experimental profiling to drive scalable and robust consortium design. Classical ecological approaches such as generalized Lotka–Volterra, consumer-resource, and trait-based models can be used to predict competitive and facilitative interactions and be expanded to incorporate more soil-relevant resource regimes [100]. Furthermore, individual-based models can add spatial realism by explicitly simulating single-cell growth, division, motility, and environmental responses to provide predictions linking localized interactions with emergent community outcomes under heterogeneous soil environments [101]. In contrast to these population- and cell-based approaches, genome-scale metabolic models simulate internal biochemical reaction networks to predict metabolic competition, complementarity, and cross-feeding, offering mechanistic insights for identifying strain combinations that can coexist with desired collective functioning [100, 102]. Integration with experimental and multi-omics data to provide regulatory and environmental constraints can further improve predictions [103]. Moreover, given the increasing availability of genomic, molecular, and single-cell data, integration across the different datasets and models will benefit from rapid development in AI including machine learning, elucidating interaction mechanisms while narrowing the vast combinatorial space of strain combinations to a tractable set for prioritized experimental testing [47]. This would strengthen the design–build–test–learn cycle for engineering consortium inoculants as ecologically stable communities with targeted functional goals and tailored to soil conditions [11, 104]. A recent study that developed the SuperCC metabolic modeling pipeline to simulate metabolic interactions and community performance is a great example illustrating the power of integrating experimental and modeling approaches for building synthetic consortia [105]. This study combined top–down and bottom–up strategies, as well as metabolic modeling and machine learning with multi-omics and isotope analyses, effectively constructing synthetic consortia based on keystone species identified from natural microbiomes with enhanced bioremediation function.

### “Cross-community” interactions between consortium inoculants and native soil microbiomes

#### Harnessing indigenous species via an integrated top–down and bottom–up strategy to overcome native community resistance

Native microbial communities can impose strong resistance against introduced consortia. Emerging evidence suggests that compatible interactions with native communities may be a determining factor in the success of inoculants. In particular, home-field advantage, which refers to microbes performing better in their native environments or historical habitats [106], should be leveraged to harness naturally occurring microbes to design tailored inoculants to promote establishment and functional performance in specific soil systems [62, 88]. This is because indigenous microbes are more adapted to the local abiotic and biotic conditions, therefore increasing the probability of their survival after inoculation. However, this needs to be balanced with potential competition between inoculants and closely related resident species with overlapping niches [25]. An integrated top–down and bottom–up strategy presents a promising path [11–13, 102]. A top–down approach can be used to enrich simplified communities with targeted functions from complex natural microbiomes, preserving the evolved ecological interaction networks that already exhibit compatibility with local soil communities [46]. By detailed profiling of these simplified communities using multi-omics methods, key taxa (e.g. core, keystone, and helper species) may be identified and isolated for characterization [38, 88, 105]. A bottom–up approach can then include these key native candidates to assemble defined communities with complementary functional roles and reduced niche overlap [107]. Together, by harnessing indigenous species, this combined strategy may increase the likelihood that the designed consortium inoculants can better establish in competitive soil environments.

#### Leveraging microbe-supporting substrates to facilitate inoculant establishment by creating niches and improving competitiveness

Classic niche-based invasion resistance theory indicates the availability of unoccupied resource niches and the capacity of invaders to utilize these resources are critical for successful establishment in resident communities. Thus, adding targeted resources can help steer the “cross-community” interactions towards facilitation by reducing competitive pressures. Prebiotics, such as metabolites that are found to promote members of the inoculant consortia or positive interactions, can be applied to provide a selective niche to enhance the growth and establishment of synthetic consortium inoculants [66, 108, 109]. Inspiration can be drawn from gut microbiome research, where prebiotics can selectively stimulate beneficial commensal bacterial colonization and establishment in gut microbiota [110]. Similarly, commercial products already exist to promote engineered microbial systems for wastewater treatment. For example, SmartBOD (Aquafix Inc.) provides a balanced blend of carbon sources to support stable microbial activity even during nutrient-limited or toxic conditions, while Foam Buster (Aquafix Inc.) can selectively promote beneficial floc-forming bacteria to outcompete problematic filamentous organisms by providing a unique blend of nutrients to improve the treatment process. As for soil applications, a recent work using sterile soil microcosms demonstrated that the provision of a selective nutrient niche (toluene) improved the proliferation of a toluene-degrading species inoculated into a standardized naturally derived soil community [55]. These examples highlight the value of tailored nutritional environments to guide microbial dynamics and enhance inoculant success, which could be translated to the soil applications of synthetic consortium inoculants.

#### Recognizing the importance of rare taxa as potential drivers of resilience in functionality

When applying synthetic inoculants, even when the dominant taxa in native communities had no significant changes, there could be shifts in the abundances of relatively rare taxa [63], which may become dominant when conditions become more favorable for them [74]. Some evidence suggests that rare taxa, rather than the dominant taxa, may be the major drivers of microbial functional resilience in soil ecosystems [73, 75]. However, rare taxa are often treated as analytical annoyances due to their low abundances. They are frequently excluded during the differential abundance analysis and overlooked in the quantitative polymerase chain reaction (qPCR) analysis due to their extreme sparsity (potentially below the limit of detection) [23, 111]. This may lead to an incomplete screening of microbial community dynamics and a failed identification of keystone taxa responsible for community stability and function under environmental shifts. To more comprehensively capture microbial community shifts, it is essential to complement abundance–occupancy metrics from amplicon sequencing with approaches such as metatranscriptomics, metaproteomics, and metabolomics. These multi-omics approaches can help reveal how rare taxa contribute to ecological processes [112]. Although it remains challenging to directly validate the traits of rare taxa by isolating them due to their highly specific environmental and nutritional requirements [112], it is possible to test their functional roles by comparing the ecological impacts of removing rare taxa through dilution-to-extinction approaches [113, 114]. Furthermore, when working with low-biomass microbiome samples near the limits of sequencing detection, it is essential to follow appropriate procedures to avoid contamination from external sources and cross-contamination [115].

### Assessing soil functional resilience in the long term

Thus far, very little is known about the long-term effects of consortium inoculants on soil resilience. Among the 104 studies of consortium inoculants in soil applications we compiled, <10 studies reported soil microbial community composition recovery toward pre-inoculation states, but none examined functional resilience (Supplementary Table S1). Time-series sampling is essential to distinguish between temporary changes and lasting shifts, enabling the identification of two critical thresholds: the point where resistance transitions into resilience and the point where resilience gives way to a tipping point in response to disturbance [116]. Using multi-omics integrated with advanced activity analyses (including isotope probing and bioorthogonal noncanonical amino acid tagging or BONCAT [117]) can connect microbial diversity, activities, functional shifts, and ecosystem performance together, providing a comprehensive understanding of microbial interactions, adaptation, and resilience. Further, more standardized long-term monitoring protocols should be developed to integrate data across molecular, population, community, and ecosystem scales. Soil resistance and resilience are governed by both “within-community” and “cross-community” interactions. Long-term assessment can help evaluate the structure and functional stability of the ecosystem, which can effectively predict the potential risks [118] and benefits of inoculations.

We hope that these proposed future research focuses emphasizing perspectives on microbial interactions will help promote more effective design and application of synthetic consortium inoculants for sustainable agriculture, climate change mitigation, and ecosystem restoration. A deeper understanding of both “within-community” and “cross-community” interactions will help optimize synthetic consortium inoculants for facilitating adaptation to different environmental conditions and maximizing their role in ecological functionality.

## Supplementary Material

Supplementary_Table_1_wrag050

## Data Availability

All data generated or analyzed during this study are included in this published article and its supplementary information file.

## References

[1] Crowther TW, Rappuoli R, Corinaldesi C. et al. Scientists' call to action: microbes, planetary health, and the sustainable development goals. *Cell* 2024;187:5195–216. 10.1016/j.cell.2024.07.051 39303686

[2] Coban O, De Deyn GB, van der Ploeg M. Soil microbiota as game-changers in restoration of degraded lands. *Science* 2022;375:abe0725. 10.1126/science.abe0725 35239372

[3] Hartmann M, Six J. Soil structure and microbiome functions in agroecosystems. *Nat Rev Earth Environ* 2023;4:4–18. 10.1038/s43017-022-00366-w

[4] Banerjee S, van der Heijden MGA. Soil microbiomes and one health. *Nat Rev Microbiol* 2023;21:6–20. 10.1038/s41579-022-00779-w 35999468

[5] Jansson JK, McClure R, Egbert RG. Soil microbiome engineering for sustainability in a changing environment. *Nat Biotechnol* 2023;41:1716–28. 10.1038/s41587-023-01932-3 37903921

[6] Silverstein MR, Segrè D, Bhatnagar JM. Environmental microbiome engineering for the mitigation of climate change. *Glob Chang Biol* 2023;29:2050–66. 10.1111/gcb.16609 36661406

[7] Jaiswal S, Shukla P. Alternative strategies for microbial remediation of pollutants via synthetic biology. *Front Microbiol* 2020;11:808. 10.3389/fmicb.2020.00808 32508759 PMC7249858

[8] Arif I, Batool M, Schenk PM. Plant microbiome engineering: expected benefits for improved crop growth and resilience. *Trends Biotechnol* 2020;38:1385–96. 10.1016/j.tibtech.2020.04.015 32451122

[9] Beattie Gwyn A, Edlund A, Esiobu N. et al. Soil microbiome interventions for carbon sequestration and climate mitigation. *mSystems* 2024;10:e01129–4. 10.1128/msystems.01129-24 39692482 PMC11748500

[10] Xu X, Dinesen C, Pioppi A. et al. Composing a microbial symphony: synthetic communities for promoting plant growth. *Trends Microbiol* 2025;33:738–51. 10.1016/j.tim.2025.01.006 39966007

[11] Lawson CE, Harcombe WR, Hatzenpichler R. et al. Common principles and best practices for engineering microbiomes. *Nat Rev Microbiol* 2019;17:725–41. 10.1038/s41579-019-0255-9 31548653 PMC8323346

[12] Lyu X, Nuhu M, Candry P. et al. Top-down and bottom-up microbiome engineering approaches to enable biomanufacturing from waste biomass. *J Ind Microbiol Biotechnol* 2024;51:kuae025. 10.1093/jimb/kuae025 39003244 PMC11287213

[13] Mehlferber EC, Arnault G, Joshi B. et al. A cross-systems primer for synthetic microbial communities. *Nat Microbiol* 2024;9:2765–73. 10.1038/s41564-024-01827-2 39478083 PMC11660114

[14] Delgado-Baquerizo M . Simplifying the complexity of the soil microbiome to guide the development of next-generation SynComs. *J Sustain Agric Environ* 2022;1:9–15. 10.1002/sae2.12012

[15] Liu X, Mei S, Salles JF. Inoculated microbial consortia perform better than single strains in living soil: a meta-analysis. *Appl Soil Ecol* 2023;190:105011. 10.1016/j.apsoil.2023.105011

[16] Kaminsky LM, Trexler RV, Malik RJ. et al. The inherent conflicts in developing soil microbial inoculants. *Trends Biotechnol* 2019;37:140–51. 10.1016/j.tibtech.2018.11.011 30587413

[17] Sessitsch A, Pfaffenbichler N, Mitter B. Microbiome applications from lab to field: facing complexity. *Trends Plant Sci* 2019;24:194–8. 10.1016/j.tplants.2018.12.004 30670324

[18] Ghoul M, Mitri S. The ecology and evolution of microbial competition. *Trends Microbiol* 2016;24:833–45. 10.1016/j.tim.2016.06.011 27546832

[19] Kost C, Patil KR, Friedman J. et al. Metabolic exchanges are ubiquitous in natural microbial communities. *Nat Microbiol* 2023;8:2244–52. 10.1038/s41564-023-01511-x 37996708

[20] Banerjee S, Schlaeppi K, van der Heijden MGA. Keystone taxa as drivers of microbiome structure and functioning. *Nat Rev Microbiol* 2018;16:567–76. 10.1038/s41579-018-0024-1 29789680

[21] Singh BK, Jiang G, Wei Z. et al. Plant pathogens, microbiomes, and soil health. *Trends Microbiol* 2025;33:887–902. 10.1016/j.tim.2025.03.013 40274492

[22] Li J, Yang C, Jousset A. et al. Engineering multifunctional rhizosphere probiotics using consortia of *bacillus amyloliquefaciens* transposon insertion mutants. *elife* 2023;12:e90726. 10.7554/eLife.90726 37706503 PMC10519709

[23] Papin M, Philippot L, Breuil MC. et al. Survival of a microbial inoculant in soil after recurrent inoculations. *Sci Rep* 2024;14:4177. 10.1038/s41598-024-54069-x 38378706 PMC10879113

[24] van Elsas JD, Chiurazzi M, Mallon CA. et al. Microbial diversity determines the invasion of soil by a bacterial pathogen. *Proc Natl Acad Sci USA* 2012;109:1159–64. 10.1073/pnas.1109326109 22232669 PMC3268289

[25] Jones ML, Ramoneda J, Rivett DW. et al. Biotic resistance shapes the influence of propagule pressure on invasion success in bacterial communities. *Ecology* 2017;98:1743–9. 10.1002/ecy.1852 28397255

[26] Liu X, Le Roux X, Salles JF. The legacy of microbial inoculants in agroecosystems and potential for tackling climate change challenges. *iScience* 2022;25:103821. 10.1016/j.isci.2022.103821 35243218 PMC8867051

[27] Li C, Chen X, Jia Z. et al. Meta-analysis reveals the effects of microbial inoculants on the biomass and diversity of soil microbial communities. *Nat Ecol Evol* 2024;8:1270–84. 10.1038/s41559-024-02437-1 38849504

[28] Mawarda PC, Le Roux X, Dirk van Elsas J. et al. Deliberate introduction of invisible invaders: a critical appraisal of the impact of microbial inoculants on soil microbial communities. *Soil Biol Biochem* 2020;148:107874. 10.1016/j.soilbio.2020.107874

[29] Oña L, Shreekar SK, Kost C. Disentangling microbial interaction networks. *Trends Microbiol* 2025;33:619–34. 10.1016/j.tim.2025.01.013 40044528

[30] Geesink P, ter Horst J, Ettema TJG. More than the sum of its parts: uncovering emerging effects of microbial interactions in complex communities. *FEMS Microbiol Ecol* 2024;100:fiae029. 10.1093/femsec/fiae029 38444203 PMC10950044

[31] Felici C, Vettori L, Giraldi E. et al. Single and co-inoculation of *Bacillus subtilis* and *Azospirillum brasilense* on *Lycopersicon esculentum*: effects on plant growth and rhizosphere microbial community. *Appl Soil Ecol* 2008;40:260–70. 10.1016/j.apsoil.2008.05.002

[32] Kehe J, Ortiz A, Kulesa A. et al. Positive interactions are common among culturable bacteria. *Sci Adv* 2021;7:eabi7159. 10.1126/sciadv.abi7159 34739314 PMC8570599

[33] Foster Kevin R, Bell T. Competition, not cooperation, dominates interactions among culturable microbial species. *Curr Biol* 2012;22:1845–50. 10.1016/j.cub.2012.08.005 22959348

[34] Emmanuel OC, Babalola OO. Productivity and quality of horticultural crops through co-inoculation of arbuscular mycorrhizal fungi and plant growth promoting bacteria. *Microbiol Res* 2020;239:126569. 10.1016/j.micres.2020.126569 32771873

[35] Zhang L, Xu M, Liu Y. et al. Carbon and phosphorus exchange may enable cooperation between an arbuscular mycorrhizal fungus and a phosphate-solubilizing bacterium. *New Phytol* 2016;210:1022–32. 10.1111/nph.13838 27074400

[36] Shrestha S, Goswami S, Banerjee D. et al. Perspective on lignin conversion strategies that enable next generation biorefineries. *ChemSusChem* 2024;17:e202301460. 10.1002/cssc.202301460 38669480

[37] Santhanam R, Menezes RC, Grabe V. et al. A suite of complementary biocontrol traits allows a native consortium of root-associated bacteria to protect their host plant from a fungal sudden-wilt disease. *Mol Ecol* 2019;28:1154–69. 10.1111/mec.15012 30633416

[38] Peng Q, Zhao C, Wang X. et al. Modeling bacterial interactions uncovers the importance of outliers in the coastal lignin-degrading consortium. *Nat Commun* 2025;16:639. 10.1038/s41467-025-56012-8 39809803 PMC11733112

[39] Xun W, Ren Y, Yan H. et al. Sustained inhibition of maize seed-borne *fusarium* using a *bacillus*-dominated rhizospheric stable core microbiota with unique cooperative patterns. *Adv Sci* 2023;10:2205215. 10.1002/advs.202205215 PMC9929125 36529951

[40] Niu B, Paulson JN, Zheng X. et al. Simplified and representative bacterial community of maize roots. *Proc Natl Acad Sci USA* 2017;114:E2450–9. 10.1073/pnas.1616148114 28275097 PMC5373366

[41] Xie L, Lehvävirta S, Timonen S. et al. Species-specific synergistic effects of two plant growth—promoting microbes on green roof plant biomass and photosynthetic efficiency. *PLoS One* 2019;13:e0209432. 10.1371/journal.pone.0209432 PMC6312232 30596699

[42] Zhang L, Zhang M, Huang S. et al. A highly conserved core bacterial microbiota with nitrogen-fixation capacity inhabits the xylem sap in maize plants. *Nat Commun* 2022;13:3361. 10.1038/s41467-022-31113-w 35688828 PMC9187771

[43] Zhu L, Wang S, Duan H. et al. Foliar pathogen-induced assemblage of beneficial rhizosphere consortia increases plant defense against *Setosphaeria turcica*. *Front Biosci (Landmark Ed)* 2021;26:543–55. 10.52586/4966 34590466

[44] Hu J, Yang T, Friman V-P. et al. Introduction of probiotic bacterial consortia promotes plant growth via impacts on the resident rhizosphere microbiome. *Proc R Soc B* 2021;288:20211396. 10.1098/rspb.2021.1396 PMC8511750 34641724

[45] Trabelsi D, Ben Ammar H, Mengoni A. et al. Appraisal of the crop-rotation effect of rhizobial inoculation on potato cropping systems in relation to soil bacterial communities. *Soil Biol Biochem* 2012;54:1–6. 10.1016/j.soilbio.2012.05.013

[46] Wang Z, Wang S, He Q. et al. Ecological design of high-performance synthetic microbial communities: from theoretical foundations to functional optimization. *ISME Commun* 2025;5:ycaf133. 10.1093/ismeco/ycaf133 40860568 PMC12373479

[47] Jing J, Garbeva P, Raaijmakers JM. et al. Strategies for tailoring functional microbial synthetic communities. *ISME J* 2024;18:wrae049. 10.1093/ismejo/wrae049 38537571 PMC11008692

[48] Bashan Y, LE d-B, Prabhu SR. et al. Advances in plant growth-promoting bacterial inoculant technology: formulations and practical perspectives (1998–2013). *Plant Soil* 2014;378:1–33. 10.1007/s11104-013-1956-x

[49] Qiu Z, Egidi E, Liu H. et al. New frontiers in agriculture productivity: optimised microbial inoculants and in situ microbiome engineering. *Biotechnol Adv* 2019;37:107371. 10.1016/j.biotechadv.2019.03.010 30890361

[50] Mallon CA, Poly F, Le Roux X. et al. Resource pulses can alleviate the biodiversity–invasion relationship in soil microbial communities. *Ecology* 2015;96:915–26. 10.1890/14-1001.1 26230013

[51] Eisenhauer N, Schulz W, Scheu S. et al. Niche dimensionality links biodiversity and invasibility of microbial communities. *Funct Ecol* 2013;27:282–8. 10.1111/j.1365-2435.2012.02060.x

[52] Mallon CA, JDV E, Salles JF. Microbial invasions: the process, patterns, and mechanisms. *Trends Microbiol* 2015;23:719–29. 10.1016/j.tim.2015.07.013 26439296

[53] Chiba A, Uchida Y, Kublik S. et al. Soil bacterial diversity is positively correlated with decomposition rates during early phases of maize litter decomposition. *Microorganisms* 2021;9:357. 10.3390/microorganisms9020357 33670245 PMC7916959

[54] Kurkjian HM, Akbari MJ, Momeni B. The impact of interactions on invasion and colonization resistance in microbial communities. *PLoS Comput Biol* 2021;17:e1008643. 10.1371/journal.pcbi.1008643 33481772 PMC7857599

[55] Čaušević S, Dubey M, Morales M. et al. Niche availability and competitive loss by facilitation control proliferation of bacterial strains intended for soil microbiome interventions. *Nat Commun* 2024;15:2557. 10.1038/s41467-024-46933-1 38519488 PMC10959995

[56] Albright MBN, Louca S, Winkler DE. et al. Solutions in microbiome engineering: prioritizing barriers to organism establishment. *ISME J* 2021;16:331–8. 10.1038/s41396-021-01088-5 34420034 PMC8776856

[57] Rivett DW, Jones ML, Ramoneda J. et al. Elevated success of multispecies bacterial invasions impacts community composition during ecological succession. *Ecol Lett* 2018;21:516–24. 10.1111/ele.12916 29446215

[58] Vila JCC, Jones ML, Patel M. et al. Uncovering the rules of microbial community invasions. *Nat Ecol Evol* 2019;3:1162–71. 10.1038/s41559-019-0952-9 31358951

[59] Hu J, Wei Z, Friman V-P. et al. Probiotic diversity enhances rhizosphere microbiome function and plant disease suppression. *MBio* 2016;7:8. 10.1128/mbio.01790-16 PMC5156302 27965449

[60] Louca S, Polz MF, Mazel F. et al. Function and functional redundancy in microbial systems. *Nat Ecol Evol* 2018;2:936–43. 10.1038/s41559-018-0519-1 29662222

[61] Eisenhauer N, Hines J, Maestre FT. et al. Reconsidering functional redundancy in biodiversity research. *npj Biodivers* 2023;2:9. 10.1038/s44185-023-00015-5 39242717 PMC11332098

[62] Jiang M, Delgado-Baquerizo M, Yuan MM. et al. Home-based microbial solution to boost crop growth in low-fertility soil. *New Phytol* 2023;239:752–65. 10.1111/nph.18943 37149890

[63] Gu Y, Meng D, Yang S. et al. Invader-resident community similarity contribute to the invasion process and regulate biofertilizer effectiveness. *J Clean Prod* 2019;241:118278. 10.1016/j.jclepro.2019.118278

[64] Wen T, Xie P, Liu H. et al. Tapping the rhizosphere metabolites for the prebiotic control of soil-borne bacterial wilt disease. *Nat Commun* 2023;14:4497. 10.1038/s41467-023-40184-2 37495619 PMC10372070

[65] Bailey C, Gwyther P, Čaušević S. et al. Phosphite as an engineered niche for *pseudomonas veronii* in a synthetic soil bacterial community. *mSystems* 2025;10:e00061–25. 10.1128/msystems.00061-25 40815466 PMC12455932

[66] Henry LP, Bergelson J. Applying ecological principles to microbiome engineering. *Nat Microbiol* 2025;10:2111–21. 10.1038/s41564-025-02076-7 40764435

[67] Chen Y, Zang H, Bai L. et al. Repeated inoculations improve wheat yield through modifying the rhizobacterial communities and nitrogen and phosphorus fractions. *Appl Soil Ecol* 2024;196:105287. 10.1016/j.apsoil.2024.105287

[68] Wang Z, Leite MFA, Jiang M. et al. Responses of soil rare and abundant microorganisms to recurring biotic disturbances. *Soil Biol Biochem* 2023;177:108913. 10.1016/j.soilbio.2022.108913

[69] Wang Z, Chen Z, Kowalchuk GA. et al. Succession of the resident soil microbial community in response to periodic inoculations. *Appl Environ Microbiol* 2021;87:e00046–21. 10.1128/AEM.00046-21 33637572 PMC8091015

[70] Johnson JS, Spakowicz DJ, Hong B-Y. et al. Evaluation of 16S rRNA gene sequencing for species and strain-level microbiome analysis. *Nat Commun* 2019;10:5029. 10.1038/s41467-019-13036-1 31695033 PMC6834636

[71] Scholz M, Ward DV, Pasolli E. et al. Strain-level microbial epidemiology and population genomics from shotgun metagenomics. *Nat Methods* 2016;13:435–8. 10.1038/nmeth.3802 26999001

[72] Jia X, Dini-Andreote F, Falcão SJ. Community assembly processes of the microbial rare biosphere. *Trends Microbiol* 2018;26:738–47. 10.1016/j.tim.2018.02.011 29550356

[73] Jousset A, Bienhold C, Chatzinotas A. et al. Where less may be more: how the rare biosphere pulls ecosystems strings. *ISME J* 2017;11:853–62. 10.1038/ismej.2016.174 28072420 PMC5364357

[74] Shade A, Jones Stuart E, Caporaso JG. et al. Conditionally rare taxa disproportionately contribute to temporal changes in microbial diversity. *MBio* 2014;5:9. 10.1128/mbio.01371-14 PMC4161262 25028427

[75] Liu Y, Tian J, Yang G. et al. Rare keystone taxa drive soil microbial stability in low-temperature peatlands of China. *Appl Soil Ecol* 2025;213:106290. 10.1016/j.apsoil.2025.106290

[76] Sun Q-w, Chen J-z, Liao X-f. et al. Identification of keystone taxa in rhizosphere microbial communities using different methods and their effects on compounds of the host *Cinnamomum migao*. *Sci Total Environ* 2024;926:171952. 10.1016/j.scitotenv.2024.171952 38537823

[77] Zhang N, Liang C, Liu X. et al. Divergent temporal response of abundant and rare bacterial communities to transient *Escherichia coli* O157:H7 invasion. *Front Microbiol* 2021;12:665380. 10.3389/fmicb.2021.665380 34163444 PMC8215281

[78] Cornell C, Kokkoris V, Richards A. et al. Do bioinoculants affect resident microbial communities? A meta-analysis. *Front Agron* 2021;3:753474. 10.3389/fagro.2021.753474

[79] Silva Batista JS, Hungria M, Barcellos FG. et al. Variability in *Bradyrhizobium japonicum* and *B. elkanii* seven years after introduction of both the exotic microsymbiont and the soybean host in a cerrados soil. *Microb Ecol* 2007;53:270–84. 10.1007/s00248-006-9149-2 17265000

[80] Sullivan JT, Patrick HN, Lowther WL. et al. Nodulating strains of *rhizobium loti* arise through chromosomal symbiotic gene transfer in the environment. *Proc Natl Acad Sci USA* 1995;92:8985–9. 10.1073/pnas.92.19.8985 7568057 PMC41092

[81] Allison SD, Martiny JBH. Resistance, resilience, and redundancy in microbial communities. *Proc Natl Acad Sci USA* 2008;105:11512–9. 10.1073/pnas.0801925105 18695234 PMC2556421

[82] Shade A . Microbiome rescue: directing resilience of environmental microbial communities. *Curr Opin Microbiol* 2023;72:102263. 10.1016/j.mib.2022.102263 36657335

[83] Philippot L, Griffiths Bryan S, Langenheder S. Microbial community resilience across ecosystems and multiple disturbances. *Microbiol Mol Biol Rev* 2021;85:24. 10.1128/mmbr.00026-20 PMC8139522 33789927

[84] Shade A, Peter H, Allison SD. et al. Fundamentals of microbial community resistance and resilience. *Front Microbiol* 2012;3:417. 10.3389/fmicb.2012.00417 PMC3525951 23267351

[85] Bagra K, Bellanger X, Merlin C. et al. Environmental stress increases the invasion success of antimicrobial resistant bacteria in river microbial communities. *Sci Total Environ* 2023;904:166661. 10.1016/j.scitotenv.2023.166661 37652387

[86] Amor DR, Ratzke C, Gore J. Transient invaders can induce shifts between alternative stable states of microbial communities. *Sci Adv* 2020;6:eaay8676. 10.1126/sciadv.aay8676 32128414 PMC7030923

[87] Mallon CA, Le Roux X, van Doorn GS. et al. The impact of failure: unsuccessful bacterial invasions steer the soil microbial community away from the invader’s niche. *ISME J* 2018;12:728–41. 10.1038/s41396-017-0003-y 29374268 PMC5864238

[88] Zhou Y, Liu D, Li F. et al. Superiority of native soil core microbiomes in supporting plant growth. *Nat Commun* 2024;15:6599. 10.1038/s41467-024-50685-3 39097606 PMC11297980

[89] Hofer U . The majority is uncultured. *Nat Rev Microbiol* 2018;16:716–7. 10.1038/s41579-018-0097-x 30275521

[90] Parks DH, Rinke C, Chuvochina M. et al. Recovery of nearly 8,000 metagenome-assembled genomes substantially expands the tree of life. *Nat Microbiol* 2017;2:1533–42. 10.1038/s41564-017-0012-7 28894102

[91] Cross KL, Campbell JH, Balachandran M. et al. Targeted isolation and cultivation of uncultivated bacteria by reverse genomics. *Nat Biotechnol* 2019;37:1314–21. 10.1038/s41587-019-0260-6 31570900 PMC6858544

[92] Huang Y, Sheth RU, Zhao S. et al. High-throughput microbial culturomics using automation and machine learning. *Nat Biotechnol* 2023;41:1424–33. 10.1038/s41587-023-01674-2 36805559 PMC10567565

[93] Li S, Lian W-H, Han J-R. et al. Capturing the microbial dark matter in desert soils using culturomics-based metagenomics and high-resolution analysis. *NPJ Biofilms Microbiomes* 2023;9:67. 10.1038/s41522-023-00439-8 37736746 PMC10516943

[94] Persyn A, Mueller A, Goormachtig S. Drops join to make a stream: high-throughput nanoscale cultivation to grasp the lettuce root microbiome. *Environ Microbiol Rep* 2022;14:60–9. 10.1111/1758-2229.13014 34797028

[95] Wang Y, Wilhelm RC, Swenson TL. et al. Substrate utilization and competitive interactions among soil bacteria vary with life-history strategies. *Front Microbiol* 2022;13:914472. 10.3389/fmicb.2022.914472 35756023 PMC9225577

[96] Jo C, Bernstein DB, Vaisman N. et al. Construction and modeling of a coculture microplate for real-time measurement of microbial interactions. *mSystems* 2023;8:e00017–21. 10.1128/msystems.00017-21 36802169 PMC10134821

[97] Chodkowski JL, Shade A. A synthetic community system for probing microbial interactions driven by exometabolites. *mSystems* 2017;2:14. 10.1128/msystems.00129-17 PMC5686522 29152587

[98] Kehe J, Kulesa A, Ortiz A. et al. Massively parallel screening of synthetic microbial communities. *Proc Natl Acad Sci USA* 2019;116:12804–9. 10.1073/pnas.1900102116 31186361 PMC6600964

[99] Hsu RH, Clark RL, Tan JW. et al. Microbial interaction network inference in microfluidic droplets. *Cell Syst* 2019;9:229–242.e224. 10.1016/j.cels.2019.06.008 31494089 PMC6763379

[100] van den Berg NI, Machado D, Santos S. et al. Ecological modelling approaches for predicting emergent properties in microbial communities. *Nat Ecol Evol* 2022;6:855–65. 10.1038/s41559-022-01746-7 35577982 PMC7613029

[101] Wang J, Hashem I, Bhonsale S. et al. Individual-based modeling unravels spatial and social interactions in bacterial communities. *ISME J* 2025;19:wraf116. 10.1093/ismejo/wraf116 40838740 PMC12411854

[102] San León D, Nogales J. Toward merging bottom–up and top–down model-based designing of synthetic microbial communities. *Curr Opin Microbiol* 2022;69:102169. 10.1016/j.mib.2022.102169 35763963

[103] Sher D, Segrè D, Follows MJ. Quantitative principles of microbial metabolism shared across scales. *Nat Microbiol* 2024;9:1940–53. 10.1038/s41564-024-01764-0 39107418

[104] Karkaria BD, Fedorec AJH, Barnes CP. Automated design of synthetic microbial communities. *Nat Commun* 2021;12:672. 10.1038/s41467-020-20756-2 33510148 PMC7844305

[105] Ruan Z, Chen K, Cao W. et al. Engineering natural microbiomes toward enhanced bioremediation by microbiome modeling. *Nat Commun* 2024;15:4694. 10.1038/s41467-024-49098-z 38824157 PMC11144243

[106] Fanin N, Fromin N, Bertrand I. Functional breadth and home-field advantage generate functional differences among soil microbial decomposers. *Ecology* 2016;97:1023–37. 10.1890/15-1263.1 27220218

[107] Troiano DT, Studer MHP. Microbial consortia for the conversion of biomass into fuels and chemicals. *Nat Commun* 2025;16:6712. 10.1038/s41467-025-61957-x 40691146 PMC12280090

[108] Vassileva M, Flor-Peregrin E, Malusá E. et al. Towards better understanding of the interactions and efficient application of plant beneficial prebiotics, probiotics, postbiotics and synbiotics. *Front Plant Sci* 2020;11:1068. 10.3389/fpls.2020.01068 32765556 PMC7378762

[109] Du J, Li Y, Ur-Rehman S. et al. Synergistically promoting plant health by harnessing synthetic microbial communities and prebiotics. *iScience* 2021;24:102918. 10.1016/j.isci.2021.102918 34430808 PMC8365361

[110] Tuohy KM, Probert HM, Smejkal CW. et al. Using probiotics and prebiotics to improve gut health. *Drug Discov Today* 2003;8:692–700. 10.1016/S1359-6446(03)02746-6 12927512

[111] Nearing JT, Douglas GM, Hayes MG. et al. Microbiome differential abundance methods produce different results across 38 datasets. *Nat Commun* 2022;13:342. 10.1038/s41467-022-28034-z 35039521 PMC8763921

[112] Chakraborty D, Jousset A, Wei Z. et al. Rare taxa in the core microbiome. *Trends Microbiol* 2025;33:727–37. 10.1016/j.tim.2025.03.002 40155212

[113] Wang J, Yu J, Pan Z. et al. Rare taxa modulate the emergence of dominants in microbial communities. *MBio* 2026;17:15. 10.1128/mbio.02598-25 PMC12802277 41288099

[114] He X, Xiao X, Wei W. et al. Soil rare microorganisms mediated the plant cadmium uptake: the central role of protists. *Sci Total Environ* 2024;908:168505. 10.1016/j.scitotenv.2023.168505 37967623

[115] Fierer N, Leung PM, Lappan R. et al. Guidelines for preventing and reporting contamination in low-biomass microbiome studies. *Nat Microbiol* 2025;10:1570–80. 10.1038/s41564-025-02035-2 40542287

[116] Ludwig M, Wilmes P, Schrader S. Measuring soil sustainability via soil resilience. *Sci Total Environ* 2018;626:1484–93. 10.1016/j.scitotenv.2017.10.043 29054651

[117] Couradeau E, Sasse J, Goudeau D. et al. Probing the active fraction of soil microbiomes using BONCAT-FACS. *Nat Commun* 2019;10:2770. 10.1038/s41467-019-10542-0 31235780 PMC6591230

[118] Ladau J, Fahimipour AK, Newcomer ME. et al. Microbial inoculants and invasions: a call to action. *Trends Microbiol* 2025;33:1064–75. 10.1016/j.tim.2025.04.018 40506296

